# Beyond Physical Disability: The Social Cognition Challenges in Quality of Life Among Multiple Sclerosis Patients

**DOI:** 10.3390/healthcare13131611

**Published:** 2025-07-04

**Authors:** Triantafyllos Doskas, Kanellos C. Spiliopoulos, Constantinos Kormas, Christos Kokkotis, Liberis Dekavallas, Anna Tsiakiri, Foteini Christidi, George D. Vavougios, Dimitrios Tsiptsios, Aspasia Serdari, Nikolaos Grigoriadis, Ioannis Iliopoulos, Konstantinos Vadikolias

**Affiliations:** 1 Department of Neurology, Athens Naval Hospital, 115 21 Athens, Greece; doskastr@gmail.com (T.D.); k.spiliopoulos@ac.upatras.gr (K.C.S.); konkormas@med.uoa.gr (C.K.); dekavallas.liberis@gmail.com (L.D.); 2 Department of Neurology, Medical School, Democritus University of Thrace, 681 00 Alexandroupolis, Greece; anniw_3@hotmail.com (A.T.); christidi.f.a@gmail.com (F.C.); iiliop@hotmail.com (I.I.); vadikosm@yahoo.com (K.V.); 3 Department of Neurology, University of Patras, 265 04 Patras, Greece; 4 Department of Physical Education and Sport Science, Democritus University of Thrace, 691 00 Komotini, Greece; ckokkoti@affil.duth.gr; 5 Department of Neurology, University of Cyprus, Nicosia 1678, Cyprus; dantevavougios@hotmail.com; 6 Third Department of Neurology, Aristotle University of Thessaloniki, 541 24 Thessaloniki, Greece; 7 Department of Child and Adolescent Psychiatry, Medical School, Democritus University of Thrace, 691 00 Komotini, Greece; aserntar@med.duth.gr; 8 Second Department of Neurology, AHEPA University Hospital, Aristotle University of Thessaloniki, 546 36 Thessaloniki, Greece; ngrigoriadis@auth.gr

**Keywords:** multiple sclerosis, quality of life, social cognition, cognitive impairment, theory of mind, emotion recognition

## Abstract

Background/Objectives: Social cognition impairment is common in multiple sclerosis (MS) and could implicate the well-being of patients by promoting difficulties in social interactions. This study investigated the relationship between social cognition and quality of life (QoL) in patients with MS (PwMSs). Methods: In total, 100 PwMSs, enrolled as per distinct criteria, underwent neuropsychological assessment using validated questionnaires and scales. To assess QoL, Multiple Sclerosis Quality of Life-54 (MSQOL-54) questionnaires, both physical and mental, were utilized. The components of social cognition were evaluated using the Reading the Mind in the Eyes Test (RMET) and the Faux Pas task. The type of MS and years since diagnosis were also recorded. Results: The RMET score (β = 0.336; *p* = 0.001) and years since diagnosis (β = −0.225; *p* = 0.017) emerged as significant predictors of physical QoL, whereas the Faux Pas score did not significantly predict MSQOL-54_PHYSICAL scores (*p* = 0.451). Both Faux Pas (β = 0.247; *p* = 0.015) and RMET scores (β = 0.221; *p* = 0.028) showed a positive association with MSQOL-54_MENTAL scores. The years since diagnosis did not significantly predict the mental component of QoL (*p* = 0.635). Conclusions: Social cognition deficits are crucial for the social functioning of patients with MS, inevitably affecting both physical and mental aspects of QoL.

## 1. Introduction

Multiple sclerosis (MS) is a chronic, immune-mediated demyelinating disease of the central nervous system, causing impairments in multiple physical domains (i.e., sensorimotor, visual, cerebellar, brainstem, and bowel/bladder), as well as deficits in cognition [1]. Neuro-cognitive impairment in MS is prevalent in up to 70% of patients, involving decline in working memory, information processing speed, learning and memory, executive functions, and social cognition [2].

Social cognition refers to a construct of various processes which define social interactions by perceiving social stimuli, as well as interpreting and generating responses to the behaviors of others [3]. The basic elements of social cognition are the Theory of Mind (ToM), emotional empathy, and social perception, including emotional expression recognition. These functions coordinate vital characteristics of social participation and could influence the quality of life (QoL) in various neurological diseases [4]. Patients with MS (PwMSs) have been associated with deficits in social cognition, as shown by poor scores in ToM and expression recognition tests [5,6]. Interactions between social cognition and QoL in MS remain under-studied, whilesome reports have implied a relationship between poor performance on social cognition tasks and worse QoL scores [7].

ToM, a fundamental aspect of social cognition, enables individuals to infer and understand the beliefs, intentions, and emotions of others [8]. Impairments in ToM can lead to difficulties in interpreting social cues, responding appropriately in social situations, and maintaining social relationships. Emotional empathy, another key component, facilitates emotional connections by allowing individuals to recognize and share the emotions of others [9]. Additionally, social perception, which involves recognizing facial expressions, body language, and social norms, plays a vital role in guiding behavior in social contexts [2,10]. Given the pivotal role of social cognition in daily functioning, its deterioration may lead to increased social isolation, reduced social support, and heightened emotional distress, all of which can negatively impact QoL [7]. Therefore, investigating the extent to which social cognition deficits contribute to QoL impairments in MS is essential for developing holistic rehabilitation strategies that address both the cognitive and psychosocial challenges faced by these patients.

The aim of this study was to investigate the relationship between social cognition and QoL in patients with MS.

## 2. Methods and Materials

### 2.1. Participants

In this cross-sectional study, PwMSs who were regularly monitored at the Neurology Department of the Athens Naval Hospital were included based on predefined eligibility criteria. All data were collected at a single time point to investigate the associations between social cognition and quality of life in patients with MS. The study protocol has previously been published [11]. Inclusion criteria were as follows: (1) confirmed MS diagnosis; (2) fluency in speaking Greek language; (3) age >17 years; (4) an education >3 years; (5) Expanded Disability Status Scale (EDSS) step ≤ 8; (6) availability to attend study visits, to follow researcher instructions, and to answer questionnaires;and (7) absence of visual problems and severe cognitive dysfunction. Accordingly, subjects were excluded in the following cases: (1) a disease relapse within at least 1 month prior to the study assessments; (2) a relapse during pregnancy; (3) use of corticosteroids; (4) the presence of other comorbidities related to fatigue; and (5) active underlying infections. Approval to conduct this study was obtained by the Hospital Local Ethical Committee of the Athens Naval Hospital (6265/15 June 2020). The study procedures were in accordance with the Declaration of Helsinki and all participants provided written informed consent.

### 2.2. Questionnaires and Scales

All participants answered questionnaires regarding their demographic characteristics under the surveillance and guidance of researchers. Types of MS, years since diagnosis, and educational level were recorded. The functional status of the examined patients was evaluated with the EDSS, collecting the evidence of eight functional systems (pyramidal, cerebellar, brainstem, sensory, visual, bowel and bladder, cerebral, and other functions of the neural system) [12]. Research material further included evaluation scales of QoL and social cognitionthat are established in the MS literature. Multiple Sclerosis Quality of Life-54 (MSQOL-54) questionnaires were used to define the QoL in study subjects [13]. MSQOL-54 comprises 54 items that are classified in 12 subcategories and 2 single-item questions. MSQOLS-54 score refers to two summary scores—a physical health-related (MSQOL-54_PHYSICAL) and a mental health-related (MSQOL-54_MENTAL) score—which are produced by weighted combinations of scale subscores. Furthermore, to assess social cognition in study participants, we used the “Reading the Mind in the Eyes” test (RMET) and the Faux Pas test [14,15,16]. In the RMET, the examined subject should choose an option that best describes feelings or thoughts about every 1 of 36 figures depicting eye expressions [14]. The Faux Pas (i.e., something that should not be said) test evaluates the affective and cognitive components of the Theory of Mind (ToM), by questioning subjects to detect faux pas in 10 short scenarios [15,16]. For each test used in the present study, there are available validation studies and normative data in Greek populations [17,18] (https://www.autismresearchcentre.com/tests/faux-pas-test-adult/, accessed on 15 August 2023). In the Greek edition of the RMET, consisting of 36 eye-region pictures, each picture was surrounded by four words that describe feelings or thoughts and the subject should choose the most appropriate. The control examination referred to the identification of the gender of the depicted person. The RMET is considered a first-order ToM test since it examines how well the subject understands the mental state behind each picture [18]. In the Greek edition of the Faux Pas test, each story was followed by six questions, which required the user to detect the faux pas, to indicate the person who committed the faux pas, to comprehend the faux pas or the false belief, and, finally, to evaluate empathy. In each scenario, two control questions were included.

### 2.3. Statistical Analysis

Descriptive and inferential statistics were performed using IBM SPSS version 29.0 (IBM Corporation, Armonk, NY, USA). Statistical significance was set at a *p*-value < 0.05. The Shapiro–Wilk test was used to examine the normality of variables. Independent samples *t*-tests were conducted to compare the scores of the RMET and the Faux Pas recognition task between the various disease type groups of patients. To determine the impact of social cognition estimates and disease duration on QoL, multivariate regression models were conducted separately for the MSQOL-54 physical and mental component scores as dependent variables. Independent variables included RMET scores, Faux Pas scores, and years since diagnosis. Model diagnostics were performed to confirm assumptions of normality, linearity, and homoscedasticity. The variance explained by each model is reported using R^2^ and adjusted R^2^ values, with statistical significance set at *p* < 0.05. The results are presented as standardized beta coefficients (β) with corresponding *p*-values. In addition, ANOVA with post hoc analysis was performed to evaluate the effect of disease duration and social cognition parameters on QoL domains.

## 3. Results

A total of 100 patients with MS were included in our study—67 with relapsing remitting MS (RRMS) and 33 with secondary progressive MS (SPMS). The demographic and clinical characteristics of the examined subjects are summarized in Table 1, including scales measurements. The mean RMET score for the RRMS group (mean = 23.88; standard deviation = 3.711) was significantly higher than that for the SPMS group (mean = 20.55; standard deviation = 4.711, *p* < 0.001), indicating a moderate effect size (Cohen’s d = 0.820; 95% CI [0.386, 1.251]). The mean Faux Pas score for the RRMS group (mean = 83.16; standard deviation = 11.353) was also higher than that for the SPMS group (mean = 79.15; standard deviation = 8.707), but this difference was not statistically significant (*p* = 0.077) and had a small effect size (Cohen’s d = 0.380; 95% CI [−0.041, 0.799]).

A multivariate regression analysis was conducted to assess the predictors of physical health-related quality of life (MSQOL-54_PHYSICAL), with Faux Pas, RMET, and years since diagnosis as independent variables (Table 2). The model accounted for 20.6% of the variance in MSQOL-54_PHYSICAL scores (R^2^ = 0.206; adjusted R^2^ = 0.182; F (3, 96) = 8.321; *p* < 0.001). The RMET score emerged as a significant predictor (β = 0.336; *p* = 0.001), indicating that higher scores on the Reading the Mind in the Eyes Test are associated with a better physical QoL. Additionally, years since diagnosis was also shown as significant (β = −0.225; *p* = 0.017), suggesting that a longer disease duration is associated with a lower physical QoL. The Faux Pas score did not significantly predict MSQOL-54_PHYSICAL scores (*p* = 0.451).

A multivariate regression analysis was performed to investigate the influence of Faux Pas and RMET scores, as well as years since diagnosis, on the mental component of QoL (MSQOL-54_MENTAL) (Table 3). The model accounted for 13.6% of the variance in MSQOL-54_MENTAL scores (R^2^ = 0.136; adjusted R^2^ = 0.109; F (3, 96) = 5.034; *p* = 0.003). Both Faux Pas (β = 0.247; *p* = 0.015) and RMET scores (β = 0.221; *p* = 0.028) emerged as significant predictors, indicating their positive association with mental QoL. In contrast, years since diagnosis did not significantly predict MSQOL-54_MENTAL scores (*p* = 0.635).

## 4. Discussion

Our study indicated that deficits in social cognition could have a negative impact on the QoL of patients with MS. The findings of this study highlight the important effect of social cognition, as measured by the RMET, on the physical aspect of QoL, and further suggest an impact of disease duration on physical health-related outcomes. Furthermore, social cognition, as assessed by both Faux Pas recognition and RMET scores, could influence the mental health-related QoL among individuals, whereas the duration since diagnosis did not significantly contribute to this aspect of QoL.

Social cognition deficits are a consistent finding in studies of PwMSs [8,19]. Dysfunction in social cognition remains less acknowledged than sensorimotor and cognitive symptoms, which have been thoroughly documented; however, the sequelae of impaired social cognition could involve PwMSs in various aspects of daily life, employment, perceived anxiety, and social interactions [20,21,22]. Alterations in the core domains of social functioning could influence the level of QoL; our findings showed that social cognition deficits could directly affect QoL. It seems that these effects might be observed in all MS populations, and future research is pivotal. Notably, the meta-analysis by Lin et al. indicated that the magnitude of impairments in ToM and facial expression recognition was not necessarily affected by heterogeneity in clinical presentation, MS type (PPMS and SPMS), or the severity of non-social cognitive dysfunction [23]. Interestingly, our findings suggested that RRMS patients performed significantly better on social cognition tasks compared to SPMS patients, particularly in terms of emotion recognition (RMET). The differences in Faux Pas recognition task performance between the groups were not statistically significant, indicating a less pronounced impact of MS type on this aspect of social cognition. It is important to note that the significant differences in RMET scores between the two groups underline the impact of MS progression on cognitive functions, with SPMS patients showing greater impairment. The small effect size and non-significant difference in the Faux Pas task suggest that while social cognition may be affected in MS, it might not differ as much between these two types of MS.

Social cognition, as a multidimensional function that encompasses various cognitive processes, was found to be crucial for the well-being of patients [7]. In our study, this was profound in the aspect of mental health, where impairments both in emotion recognition and in the affective and cognitive elements of ToM indicated a poorer mental QoL. Deficits in social cognition highlight the interplay between cognitive function and social processes, impeding the interpretation of social cues and preventing communication with others. Hence, the combination of diminished social cognition and the burden of a chronic disease like MS could lead to social isolation and poor mental QoL [24]. Given the motor symptoms of the disease as well, the duration of the disease, i.e., years since diagnosis, is a significant factor of patient-reported outcomes (PROs). In terms of QoL, it seems that a longer disease duration induces a greater feeling of physical disabilities. Interestingly, impairments in social cognition were also shown to negatively affect the physical component of QoL, indicating additional difficulties in daily tasks. Hence, cognitive impairment could be involved in multiple aspects of disability in MS and should be considered when analyzing PROs. Estimates of social cognition should be included in clinical assessments of MS as valuable tools to holistically measure disability. Certain questionnaires and scales measuring social cognition could also be implemented in the long-term monitoring of MS patients, indicating the necessity to intervene with social support programs.

Our results are in agreement with previous studies suggesting a direct influence of social cognition impairments on patients’ QoL [25,26]. Specifically, Phillips and colleagues showed associations between impaired emotion perception and deficits in social and psychological QoL [25], whereas the work by Isernia et al. indicated an important implication of the affective component of ToM on both mental and physical QoL [26]. However, it should be noted that findings from previous studies remain controversial, since both studies by Grothe et al. [27] and Crivelli et al. [28] failed to detect significant relationships between social cognition and QoL [7]. Since the number of studies investigating the relationship between social cognition and QoL in MS remains limited and there is no absolute agreement between findings, our study including a large cohort of MS patients could provide valuable indications for the association of social cognition deficits with a poorer QoL. This relationship was characterized by the effect of the affective component of ToM on both the physical and mental aspects of QoL. Notably, social cognition impairment was more extensively associated with a poorer mental QoL. Overlapping symptoms of fatigue, depression, and anxiety may impede social interactions and participation in daily activities, hence complicating the investigation of the role that social cognition has in social functioning [11]. Therefore, the causative complexity of the challenges experienced by MS patients in social interactions remains to be further studied, with focus on developing efficient psychosocial programs to support patients [29].

The implications of these findings extend beyond the clinical assessment of MS-related cognitive dysfunction. The observed association between social cognition and QoL suggests that cognitive rehabilitation programs could incorporate interventions that specifically target social cognition skills. Traditional neuropsychological rehabilitation approaches often emphasize memory, attention, and executive functioning, but there is an increasing need to develop structured training programs aimed at enhancing emotion recognition, ToM, and social communication abilities. Furthermore, integrating social cognition assessments into routine MS evaluations may help clinicians identify patients at a higher risk of social withdrawal and psychological distress. By addressing these impairments early, healthcare professionals can facilitate better social reintegration, improve interpersonal relationships, and ultimately enhance patients’ overall well-being. Future research, including longitudinal and/or interventional studies, should explore whether targeted interventions, such as social cognition training or virtual reality-based social interaction programs, could serve as effective tools for improving QoL in MS patients.

It is important to consider that RMET performance may be affected by visual impairment, which is common in MS. Although we excluded individuals with severe visual dysfunction, subtle visual disturbances, such as those from prior optic neuritis or oculomotor issues, could still influence RMET scores. While the EDSS captures visual system involvement, we did not analyze the specific correlations between the EDSS visual subscale and RMET or Faux Pas scores. Future studies should consider integrating detailed visual assessments or controlling for visual acuity and oculomotor function when interpreting RMET performance in PwMSs.

Some limitations should be considered regarding this study, given its cross-sectional design. Social cognition, particularly the domains of ToM and facial emotion recognition, have been shown to be affected by impairments in various cognitive functions including IPS, working memory, and executive functions [2]. However, in this study, we examined the effect of social cognition deficits on QoL, restricting investigations for other confounding factors. The exclusion of these confounding factors could impede the causal interpretation of our findings, since these variables could influence the relationship between social cognition and QoL. Hence, future research could be controlled for the co-existence of various confounding factors. Another limitation could be the interpretation of RMET scores as a tool in MS, given the potential deficits in visual or oculomotor function. Furthermore, for the purposes of our study, the examined MS population was not discriminated based on MS type or on gender. Finally, investigations of social cognition and QoL focused on MS subjects without including a healthy control group.

## 5. Conclusions

Deficits in social cognition of PwMSs could be directly associated with impairments in both physical and mental aspects of QoL. Future research is necessary in order to provide more robust insights, incorporating further neuropsychological and neuro-imaging data. In the era of advancements in more targeted medications to treat physical symptoms, the development of efficient social interventions is also crucial for preserving patients’ well-being.

## Figures and Tables

**Table 1:** Demographics and clinical characteristics of the study subjects.

	MS Subjects *n* = 100
Gender, male/female	39/61
Age, years; median (range)	47 (20–70)
Type of MS, *n*	
Relapsing remitting	67
Secondary progressive	33
Years since diagnosis, mean (sd)	11.44 (7.72)
EDSS step, median (range)	3 (1–7)
EDSS ≤ 3.5, *n*	72
EDSS > 3.5, *n*	28
MSQOL-54, mean (sd)	
Physical component	69.03 (21.58)
Mental component	72.25 (20.14)
RMET, mean (sd)	22.78 (4.34)
Faux pas, mean (sd)	81.84 (10.68)

EDSS: Expanded Disability Status Scale; MS: multiple sclerosis; MSQOL-54: Multiple Sclerosis Quality of Life 54; RMET: Reading the Mind in the Eyes Test; sd: standard deviation.

**Table 2:** Multivariate regression analysis predicting physical health-related quality of life.

Variable	B	SE	β	t(96)	*p*	Zero-Order	Partial	Part
Constant	26.250	17.430	-	1.506	0.135	-	-	-
Faux Pas	0.146	0.192	0.072	0.757	0.451	0.197	0.077	0.069
RMET	1.670	0.473	0.336	3.531	**0.001**	0.386	0.339	0.321
Years since diagnosis	−0.628	0.258	−0.225	−2.433	**0.017**	−0.279	−0.241	−0.221

Statistical significance at *p* < 0.05 (bold). RMET: Reading the Mind in the Eyes Test.

**Table 3:** Multivariate regression analysis predicting mental health-related quality of life.

Variable	B	SE	β	t(96)	*p*	Zero-Order	Partial	Part
Constant	9.492	16.968	-	0.559	0.577	-	-	-
Faux Pas	0.465	0.187	0.247	2.482	**0.015**	0.301	0.246	0.235
RMET	1.024	0.460	0.221	2.224	**0.028**	0.283	0.221	0.211
Years since diagnosis	0.120	0.251	0.046	0.476	0.635	−0.019	0.049	0.045

Statistical significance at *p* < 0.05 (bold). RMET: Reading the Mind in the Eyes Test.

## Data Availability

All data are available upon request.
